# Manuka honey as a multifunctional therapeutic against *Helicobacter pylori*: from chemical profiling to in vitro potency and in vivo gastroprotective efficacy

**DOI:** 10.1186/s12906-026-05491-3

**Published:** 2026-08-06

**Authors:** Wafaa Maher Abd Elaziz, Eman E. Abd Elhady, Mai Hussein Abd-Elfatah, Mohammed Yosri

**Affiliations:** 1 Nutrition and Food Science Department, Faculty of Home Economics, Al-Azhar University, Cairo, Egypt; 2 The Regional Center for Mycology and Biotechnology, Al-Azhar University, Nasr City, Cairo, 11787 Egypt

**Keywords:** Manuka honey, *Helicobacter pylori*, Antibacterial, Gastroprotection, Inflammatory cytokines, Oxidative stress markers

## Abstract

Manuka honey contains bioactive phenolic and flavonoid chemicals with antibacterial and anti-inflammatory effects. The chemical profile and therapeutic efficiency of Manuka honey against *Helicobacter pylori* infection were investigated utilizing *in-vitro* and *in-vivo* models. The phenolic and flavonoid contents were profiled using HPLC. The antibacterial property of Manuka honey extract was determined using inhibitory zones, MIC, MBC, and time-kill tests. An *H. pylori* rat model was utilized to investigate changes in weight, bacterial burden, histological modifications, NF-κB expression, inflammatory cytokines (TNF-α, IL-4), oxidative stress indicators (CAT, SOD, MDA, NO), and biochemical parameters such as kidney and liver functioning. HPLC examination identified seven phenolic chemicals, the most prevalent of which were coumaric, cinnamic, and chlorogenic acids, as well as seven flavonoids, dominated by chrysin, rutin, and kaempferol. Manuka honey demonstrated strong anti-*H. pylori* activity (inhibition zone 15.1 mm), equivalent to conventional medications with MIC 31.25 µg/mL and MBC 62.5 µg/mL. Time-kill curves revealed no significant differences from normal therapy. Manuka honey treated infected rats by restoring body weight, reducing stomach bacterial load, improving histology, and downregulating NF-κB expression. The treated rats showed decreased TNF-α, increased IL-4, improved antioxidant enzyme levels, lower oxidative stress indicators, and normalized liver and kidney functioning. Manuka honey has substantial anti-*H. pylori* capacity, substantial anti-inflammatory properties, oxidative stress regulation, and protection of gastric tissue, hepatic, and renal functions, indicating its therapeutic usefulness as a natural complementary to *H. pylori* infection.

**Supplementary Information:**

The online version contains supplementary material available at 10.1186/s12906-026-05491-3.

## Introduction

Manuka honey, obtained from the nectar of *Leptospermum scoparium*, is gaining scholarly interest due to its high concentration of bioactive substances including flavonoid and other phenolic components [1, 2]. These chemicals are linked to its well-established antibacterial, anti-inflammatory, anti-oxidant, and wound-healing effects. Unlike other honeys, Manuka honey has higher antibacterial power and durability, making it a viable natural treatment agent for pathogenic and inflammatory health conditions [3, 4]. Methylglyoxal (MGO), derived from dihydroxyacetone in Manuka nectar, is the key ingredient that gives Manuka honey its powerful and stable antibacterial power; its concentration determines honey's medicinal strength, making it successful for wound healing, digestion, and antimicrobial activity, though high levels are being studied for potential risks [5].

*Helicobacter pylori* (*H. pylori*) continue to constitute an enormous world health concern as a chronic gastrointestinal pathogen linked to gastritis, peptic ulcer condition, mucosa-related lymphoid tissue (MALT) lymphoma, and gastric cancer [6]. Emerging resistance to antibiotics has drastically lowered the effectiveness rates of traditional triple- or quadruple treatment approaches, necessitating looking for new or complementary approaches to treatment [7, 8]. Natural bioactive compounds with multifactorial modes of action, such as antibacterial, immunomodulatory, and antioxidant activities, are currently being extensively studied for their possible protective functions towards *H. pylori*-associated stomach damage [9, 10]. Recent reviews have emphasized the growing role of natural products, including honey and plant-derived compounds, as alternative or adjunctive therapies against *H. pylori* infection, particularly in the context of rising multidrug resistance [9].

Manuka honey's medicinal potential versus *H. pylori* necessitates rigorous evaluation through chemical and biologic analysis. High-performance liquid chromatography (HPLC) is a precise analytical platform for detecting important phytochemical ingredients while additionally guaranteeing the consistency and uniformity of honey utilized in medicinal studies [11–13]. *In *in vitro experiments that include minimum inhibitory concentration (MIC) assessment and time-kill assessment provide critical information about the direct antibacterial consequences of Manuka honey on *H. pylori* growth characteristics [14, 15]. An in vivo* H. pylori* infection model is necessary to assess the therapeutic effectiveness along with the safety of Manuka honey under biochemical conditions in order to validate these outcomes in a biological setting. A thorough evaluation of both therapeutic possibilities and systemic security is provided by key outcomes, such as changes in body weight, stomach bacterial load, histological abnormalities, NF-κB expression, inflammatory levels of cytokines, reactive oxygen species biomarkers, and important organ function [16–18]. Previous in vitro investigations have demonstrated that Manuka honey exhibits direct antibacterial activity against *H. pylori*, attributed largely to methylglyoxal and phenolic constituents capable of disrupting bacterial membranes and interfering with microbial metabolism [3, 19, 20]. Beyond its antimicrobial action, Manuka honey has shown significant anti-inflammatory and antioxidant effects in experimental gastric injury models. In rat models of acetic acid–induced gastric ulceration, Manuka honey reduced mucosal damage, suppressed NF-κB activation, decreased TNF-α expression, and enhanced antioxidant defenses such as SOD and CAT [11, 21], Given that *H. pylori*–associated gastric pathology is strongly mediated through NF-κB–dependent inflammatory cascades and oxidative stress pathways [22, 23], these previous findings provide mechanistic justification for evaluating Manuka honey in an in vivo* H. pylori* infection model. Despite previous reports on the antibacterial activity of Manuka honey, no single study has comprehensively integrated HPLC phytochemical profiling, in vitro time-kill kinetics, and in vivo multi-parameter assessment (histopathology, inflammatory cytokines, oxidative stress, and organ function) in a single experimental model. Furthermore, the comparative efficacy of Manuka honey versus standard triple therapy in an *H. pylori*-infected rat model has not been systematically evaluated. Therefore, the present study tested the hypothesis that Manuka honey exerts dose-dependent anti-*H. pylori* activity through both direct bactericidal effects and indirect anti-inflammatory/antioxidant mechanisms. Accordingly, the current study was designed to: (i) use HPLC to determine the phytochemical profile of Manuka honey; (ii) assess its ability to fight *H. pylori* and determination of MIC as well as time-kill kinetics; and (iii) evaluate its therapeutic, anti-inflammatory, antioxidant, and gastroprotective properties in an in vivo *H. pylori*-induced gastric infection model in comparison to a standard anti-*H. pylori* medication.

## Methods and materials

### Honey specimen acquisition and processing & bacterial strain

Registered Manuka honey (MGO 550 +, New Zealand, UMF 15** + **-graded; Batch: 24-15P, Richlands QLD 4077 Australia) was purchased from an approved commercial provider. According to the manufacturer's labeling, the methylglyoxal (MGO) content is ≥ 550 mg/kg (equivalent to UMF 15 +). For experimental use, the honey was standardized by dissolving lyophilized honey in sterile distilled water to achieve stock solutions of 100 mg/mL (for in *vitro* assays) and 32 mg/mL or 64 mg/kg body weight (for in vivo administration). All solutions were freshly prepared on the day of use and filter-sterilized through 0.22 µm membranes. The specimens were kept at 4 °C in light-resistant containers. Prior to analysis or delivery, honey was brought to ambient temperature and dissolved in sterile distilled water to the relevant experimental doses [20]. *H. pylori* (ATCC 43504) was kindly provided from The Regional center for Mycology and Biotechnology, Al-Azhar University.

### HPLC testing of chemical constituents

Manuka honey specimen has been dissolved in a methanol–water solution, vortexed, filtered using a 0.22 µm membrane, and spun. The resultant supernatant was utilized for chromatographic examination. HPLC analysis was carried out on a reverse-phase C18 column. The mobile phase was made up of solvents A (water containing 0.1% formic acid) and B (acetonitrile). A gradient elution program was used at the rate of flow of 1.0 mL/min, and monitoring wavelengths specific to phenolic and flavonoid chemicals were used. Standardized calibration charts for corresponding phenolic molecules were generated. Peaks were detected through contrasting retention periods and UV spectrum, and levels were calculated via peak area correlation [24, 25]. The HPLC system consisted of an Agilent 1260 Infinity II series equipped with a C18 reversed-phase column (Agilent Zorbax Eclipse Plus, 4.6 × 250 mm, 5 µm particle size). The column temperature was maintained at 30 °C. The mobile phase comprised solvent A (water with 0.1% formic acid) and solvent B (acetonitrile with 0.1% formic acid) at a flow rate of 1.0 mL/min. The gradient program was: 0–5 min, 10% B; 5–20 min, 10–40% B; 20–30 min, 40–60% B; 30–35 min, 60–10% B; followed by re-equilibration for 5 min. Detection wavelengths were 280 nm for phenolic compounds and 360 nm for flavonoids. The injection volume was 20 µL. Method validation parameters are summarized in (Supplementary Table S1), including limit of detection (LOD; 0.05–0.21 µg/mL), limit of quantification (LOQ; 0.15–0.63 µg/mL), and recovery percentages (92.3–104.7%). All standards had purity ≥ 98% (Sigma-Aldrich, USA).

### Detection of anti.* H. pylori *via agar diffusion assay

Manuka honey extract was tested against *H. pylori* using the agar-well diffusion method with Mueller–Hinton (MH) medium supplemented with 5% defibrinated sheep blood. The inoculum was adjusted to McFarland standard No. 2 (approximately 1 × 10^8^ CFU/mL). Using a sterile cotton swab, the *H. pylori* suspension was evenly spread onto the surface of the agar plates. The test concentration of 100 mg/mL was selected based on preliminary range-finding experiments (testing 12.5, 25, 50, 100, and 200 mg/mL), which demonstrated that 100 mg/mL produced consistent, measurable inhibition zones (12–18 mm) without causing agar discoloration or precipitate formation. This concentration is also consistent with previously published studies on honey against *H. pylori* [19, 20]. To prepare the extract, lyophilized Manuka honey was dissolved in sterile distilled water to achieve a final concentration of 100 mg/mL, vortexed for 30 s, and filtered through a 0.22 µm syringe filter. Then, 100 µL of this extract was added to 6 mm diameter wells punched into the agar. Clarithromycin (0.05 μg/mL) was used as a positive control; this concentration was selected based on the CLSI breakpoint for *H. pylori* susceptibility (MIC ≤ 0.25 μg/mL), and 0.05 μg/mL represents a sub-MIC concentration that allows comparison with honey's bactericidal rather than merely bacteriostatic effects. Sterile distilled water served as the negative control (solvent control), replacing the previously used DMSO because honey is water-soluble. Plates were kept at 4 °C for 30 min to allow sample diffusion before incubation. The plates were then incubated for 72 h in a microaerobic atmosphere (85% N₂, 10% CO₂, 5% O₂) at 37 °C using a GasPak™ anaerobic system with microaerophilic sachets (Oxoid). The resulting inhibition zones were measured in millimeters [26]. All tests were performed in triplicate.

### Determination of MIC and MBC for Manuka honey versus *H. pylori*

To find the specimen's minimum inhibitory concentrations (MICs) towards *H. pylori*, Mueller–Hinton broth was utilized as the micro-dilution broth. The honey sample was serially diluted in a range of 0.98 to 1000 μg/mL. 200 μL of every specimen dilution was added to each well of a 96-well microtiter plate. To obtain the necessary McFarland turbidity of 1.0, fresh *H. pylori* inoculum was produced in sterile NaCl (0.85%). Each well received 2.0 μL of the tested organisms, resulting in an ultimate dose of 5.0 × 10^4^ colony-forming units/mL. For 72 h, the plates underwent incubation at 36 °C. After that, the MIC was visually evaluated to see how well the examined microorganism's overall development was suppressed. Every microplate that included an *H. pylori* inoculum with no the honey specimen served as a positive reference, while the microplates that contained the honey specimen without *H. pylori* served as a negative reference [27]. The micro-dilution plates used for the MIC test revealed the MBC. Each well's 10 μL aliquots that showed no signs of growth were moved to Mueller–Hinton agar plates and cultured for 72 h at 37 °C. The MBC is the smallest amount of the honey specimen that completely stopped the development of *H. pylori* on the plates. The MBC/MIC ratio was used to determine if the material possessed bacteriostatic or bactericidal properties [28].

### Testing time killing kinetics of Manuka honey versus *H. pylori*

The rate of kill kinetics of the examined honey was ascertained using a modified technique described by Appiah et al. [29]. *H. pylori* was adjusted to the McFarland standard of 0.5 following subculturing. Sterile broth was added to test tubes at levels that matched, doubled, and quadrupled the MIC of the sample under investigation. The test tubes were allowed to incubate at 37 °C after an inoculum size of 1.0 × 10^6^ cfu/mL of the established bacterium was added. A portion (1.0 mL) of the medium used were extracted and carefully injected into media in sterile Petri plates at frequencies of 0, 30, 60, 120, 150, and 180 min. The inoculum-loaded Petri dishes underwent incubation for 24 h at 38 °C after the agar had solidified. Furthermore, an assessment of control was carried out specifically for the bacteria. The procedure was repeated three times, and the colony-forming unit (CFU) of the test organisms was determined. All CFU counts were converted to Log₁₀ CFU/mL for statistical analysis. Bactericidal activity was defined as a ≥ 3 Log₁₀ reduction in CFU/mL compared to the initial inoculum. Data are presented as mean Log₁₀ CFU/mL ± standard error of the mean (SEM) from three independent experiments. Two-way repeated-measures ANOVA was used to compare the killing kinetics between Manuka honey and standard drugs, with time (0, 30, 60, 120, 150, 180 min) as the within-subjects factor and treatment (MH vs. SD) as the between-subjects factor. Tukey's post-hoc test was applied for pairwise comparisons at each time point. A *p*-value < 0.05 was considered statistically significant.

### In vivo* H. pylori *infection model

Fifty (*n* = 10 per group) adult male Wistar albino rats (120–140 g) were obtained from the Animal House Facility, Al-Azhar University, Cairo, Egypt. The sample size (*n* = 10 per group) was calculated using G*Power software (α = 0.05, power = 0.80) based on preliminary data on gastric bacterial load reduction. The animals were housed under controlled environmental conditions with a 12-h light/dark cycle, regulated temperature and humidity. The experiment began after a one-week acclimatization period. Five groups of animals (n = 10 each) were randomly selected: (1) uninfected, untreated (negative control); (2) *H. pylori*-infected, untreated (positive control); (3) *H. pylori*-infected, treated with low-dose (32 mg/kg) Manuka honey; (4) *H. pylori*-infected, treated with high-dose Manuka honey (64 mg/kg); and (5) *H. pylori*-infected, treated with standard reference drug combination (amoxicillin 102.80 mg/kg, clarithromycin 51.37 mg/kg, omeprazole 2.035 mg/kg) [30]. Successful *H. pylori* infection was confirmed on day 7 post-inoculation by collecting gastric biopsies from two rats per group (sacrificed for this purpose) and culturing on selective *H. pylori* agar under microaerobic conditions at 37 °C for 72 h. Only animals with confirmed infection (positive culture) continued in the study; the additional rats initially included (total 60) allowed for this confirmation without reducing final group sizes to below *n* = 10. The infection protocol was: *H. pylori* 1 × 10^6^ CFU/mL in 0.5 mL phosphate buffer solution (PBS) were administered orally to the animals twice a day for two days until the infection was verified [31, 32]. Treatment was orally given once daily for four weeks of time once infection was confirmed [33, 34]. Throughout the study, a weekly body weight check was conducted [35]. All animal procedures were conducted in accordance with the ARRIVE 2.0 guidelines and the National Institutes of Health Guide for the Care and Use of Laboratory Animals. For invasive procedures and terminal sample collection, rats were anesthetized using inhalational isoflurane (induction at 4–5% and maintenance at 1.5–2% in oxygen) delivered via a calibrated vaporizer system. Adequate depth of anesthesia was confirmed by loss of pedal withdrawal and corneal reflexes prior to tissue collection. At the end of the experimental period, animals were euthanized by isoflurane overdose (5% in oxygen) followed by cervical dislocation as a secondary physical method to ensure death. This protocol was approved by the Faculty of Home Economics, Al-Azhar University Research Ethics Committee (Approval No. FC01102024).

### Detection of gastric bacterial load (CFU count)

Tissues from the stomach were removed, blended after (0, 7, 14, 21, and 28 days), and serially diluted. Dilutions were cultured in a microaerophilic environment after being plated on selective agar. The number of colony-forming units (CFU) per milligram of tissue was calculated [32].

### Histopathological testing and NF-κB immunostaining

Gastric specimens have been sliced at the end of the experiment at a thickness of 4 µm after being fixed in 10% formalin and placed in paraffin. Hematoxylin and eosin (H&E) was used to stain the sections. The integrity of the stomach mucosa, inflammatory infiltration, and morphological modifications were evaluated [36]. After incubating sections. Exposing the target protein (NF-κB) for antibody binding then use a secondary antibody coupled with an enzyme (HRP), then use Mayer's Hematoxylin for coloring the nuclei. Digital evaluation of images was used to quantify the intensity of immunopositive staining [37]. Histological assessment was performed using the Updated Sydney System for semi-quantitative scoring of gastric mucosa [36]. Four parameters were evaluated: neutrophilic activity (intraepithelial neutrophils), chronic inflammation (mononuclear cell infiltration), glandular atrophy (loss of appropriate gastric glands), and surface epithelial damage. Each parameter was scored on a scale of 0 to 3 (0 = normal, 1 = mild, 2 = moderate, 3 = marked) by a pathologist blinded to the treatment groups.

### Determination of inflammatory markers

Tumor necrosis factor-alpha (TNF-α) and interleukin-4 (IL-4) were quantified at the end of the experimental model using ELISA based on the serum of rats from different testing groups, according to the instructions given by the manufacturer (Invitrogen, USA) [38].

### Assessment of oxidative stress biomarkers

Oxidative indicators in stomach tissue homogenates from rats from various tested groups were examined at the end of the experimental model using standard biochemical assays (Elabscience kits, USA): Superoxide dismutase (SOD) activity, Catalase (CAT) activity, Malondialdehyde (MDA) levels, and the quantity of Nitric oxide (NO) [39].

### Liver and kidney functions tests

In order to evaluate the systemic security of the therapies, serum biochemical markers including alanine aminotransferase (ALT), aspartate aminotransferase (AST), alkaline phosphatase (ALP), urea, and creatinine were determined at the end of the experimental model using kits from Novus Biologicals, USA, in accordance with the instruction manual [40].

### Statistical testing

Every test was carried out three times. Mean ± standard error of the mean (SEM) was used to express the data. GraphPad Prism (version 8; USA) was used to conduct statistical analyses. The Shapiro–Wilk test was used to determine whether the data distribution was normal. One-way examination of variance (ANOVA) was used for group comparisons, and Tukey's post hoc test was used to find significant pairwise variations. Two-way repeated-measures ANOVA was employed in body weight when measurements that were repeated were assessed over time. Statistical significance was defined as a *p*-value of less than 0.05.

## Results

### HPLC profiling of phenolic and flavonoid constituents

**Fig. 1:**
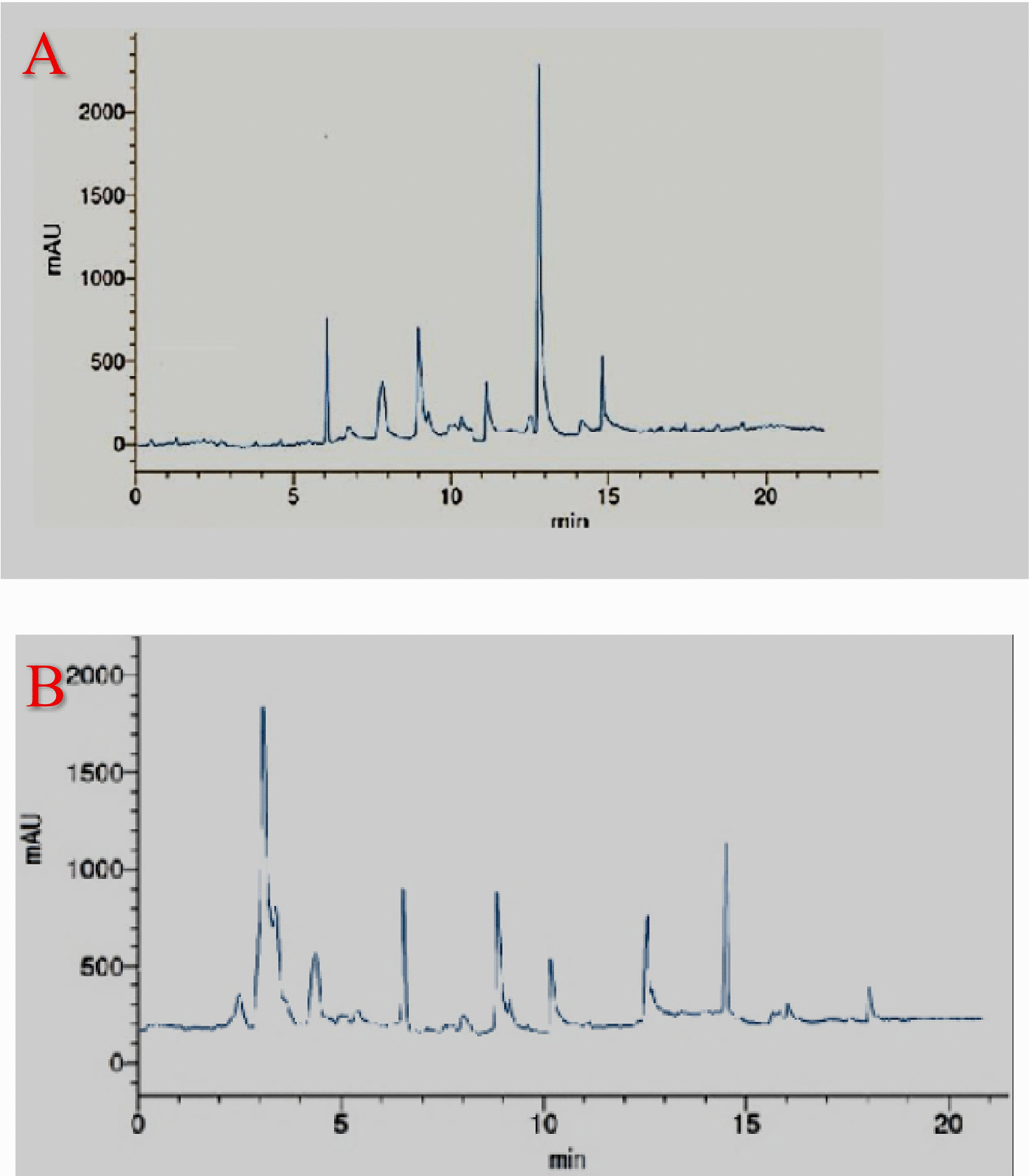
Quantitative testing for various compounds in extract of Manuka honey (**A**) Phenolic compounds; **B** Flavonoids

Quantitative testing for phenolic and flavonoid in Manuka honey has been done using HPLC (Fig. 1A, B). Seven phenolic compounds could be detected which were chlorogenic acid, cinnamic acid, ellagic acid, ferulic acid, gallic acid, coumaric acid, syringic acid. It could be noticed that coumaric acid, cinnamic acid, and chlorogenic acid were the most prevalent compounds, respectively. On the other hand, testing the flavonoids content revealed the existence of seven molecules which were: chryin**,** apigenin, catechin**,** kaempferol**,** galangin**,** luteolin, and rutin. It could be noticed that chryin, rutin, and kaempferol were the most common molecules, subsequently (Tables 1 and 2).

**Table 1:** Detected phenolic compounds and their corresponding levels in Manuka honey extract

Retention time	Compound name	Conc. (mg/mL)
5.0	Chlorogenic acid	6.44
6.0	Cinnamic acid	9.60
8.0	Ellagic acid	5.23
9.0	Ferulic acid	7.85
11.0	Gallic acid	4.30
13.0	Coumaric acid	28.70
15.0	Syringic acid	6.09

**Table 2:** Determined flavonoids and their concentrations in Manuka honey extract

Retention time	Compound name	Conc. (mg/mL)
3.0	Chryin	19.52
4.2	Apigenin	4.62
6.3	Catechin	9.51
9.0	Kaempferol	11.14
10.3	Galangin	5.20
12.8	Luteolin	6.73
14.8	Rutin	13.81

### Comprehensive assessment of anti-*H. pylori* efficacy

**Fig. 2:**
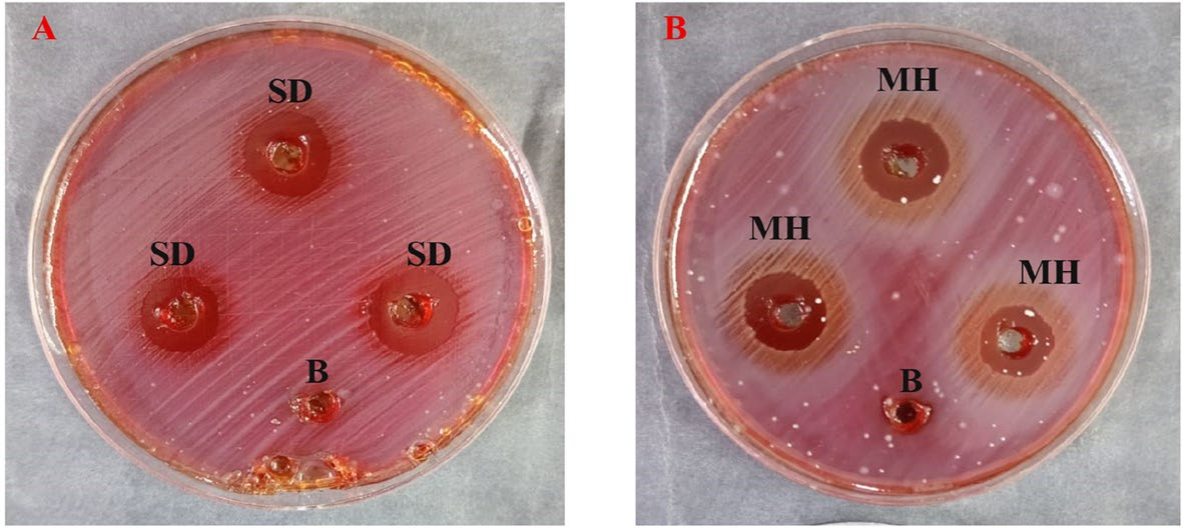
Agar diffusion assay for examination of anti-*H. pylori* for Manuka honey (MH) compared to standard drugs (SD). B: Blank

Manuka honey extract had a promising anti-*H. pylorus* with an inhibition zone = 15.1 ± 0.2 mm relative to standard drugs which had inhibition zone = 14.9 ± 0.3 mm (Fig. 2, Table 3). The MIC level for Manuka honey extract was determined at a level = 31.25 ± 0.2 µg/mL while, its MBC was detected at a concentration = 62.5 ± 0.4 µg/mL reelected its bactericidal impact of the honey extract relative to same impact detected by the standard drugs (Table 3).

**Table 3:** Testing anti-*H. pylori*, MIC, and MBC for Manuka honey (MH) compared to standard drugs (SD) (*n* = 3 independent replicates for in vitro data; Data are presented as means ± SEM)

Samples	Inhibition zone (mm)	MIC (µg/mL)	MBC (µg/mL)	MBC/MIC
SD	14.9 ± 0.3	7.8 ± 0.3	15.62 ± 0.2	2
MH	15.1 ± 0.2	31.25 ± 0.2	62.5 ± 0.4	2

### Time-kill kinetics outcomes

The time-kill kinetics of Manuka honey (MH) and standard drugs (SD) against *H. pylori* are presented in Table 4. Both treatments exhibited a time-dependent reduction in bacterial counts, with no significant difference between MH and SD at any time point (*p* > 0.05 for all comparisons). The initial inoculum was 6.00 ± 0.00 Log₁₀ CFU/mL. After 30 min, counts decreased to 5.34 ± 0.02 Log₁₀ CFU/mL for MH and 5.35 ± 0.02 Log₁₀ CFU/mL for SD. By 120 min, both treatments achieved a ≥ 2.5 Log₁₀ reduction (MH: 3.33 ± 0.02; SD: 3.35 ± 0.01 Log₁₀ CFU/mL). The bactericidal threshold (≥ 3 Log₁₀ reduction) was reached by 150 min for both treatments (MH: 1.99 ± 0.04; SD: 2.01 ± 0.03 Log₁₀ CFU/mL). Complete killing (no detectable bacteria; limit of detection = 10 CFU/mL) was achieved by 180 min in both groups. Two-way repeated-measures ANOVA confirmed no significant difference between the two treatments (F(1,4) = 0.23, p = 0.657) and no significant treatment × time interaction (F(5,20) = 0.18, *p* = 0.968), indicating that Manuka honey exhibits bactericidal kinetics equivalent to standard antibiotic therapy. A significant within-subjects effect of time (F(5,20) = 1245.6, *p* < 0.0001) confirmed the time-dependent nature of bacterial killing.

**Table 4:** Time-kill kinetics of Manuka honey (MH) compared to standard drugs (SD) against *H. pylori* ATCC 43504. Data are presented as mean Log₁₀ CFU/mL ± SEM (*n* = 3 independent experiments). Bactericidal activity is defined as a ≥ 3 Log₁₀ reduction from the initial inoculum (Time 0 = 6.00 ± 0.00 Log₁₀ CFU/mL). Values with the same superscript letter within the same row indicate no significant difference between SD and MH at that time point (two-way repeated-measures ANOVA with Tukey's post-hoc test, *p* > 0.05 for all comparisons)

Time (min)	Standard drugs (SD)	Manuka honey (MH)
	**Log₁₀ CFU/mL ± SEM**	**Log₁₀ CFU/mL ± SEM**
0	6.00 ± 0.00^a^	6.00 ± 0.00^a^
30	5.35 ± 0.02^a^	5.34 ± 0.02^a^
60	4.39 ± 0.02^a^	4.38 ± 0.03^a^
120	3.35 ± 0.01^a^	3.33 ± 0.02^a^
150	2.01 ± 0.03^a^	1.99 ± 0.04^a^
180	0.00 ± 0.00^a^	0.00 ± 0.00^a^

^*^ Statistical summary:

- Two-way repeated-measures ANOVA: No significant main effect of treatment (F (1,4) = 0.23, *p* = 0.657)

- No significant treatment × time interaction (F (5, 20) = 0.18, *p* = 0.968)

- Within-subjects effect of time: Significant (F(5, 20) = 1245.6, *p* < 0.0001), confirming time-dependent killing

### Assessment the change in weights among animals in various examined groups

**Fig. 3:**
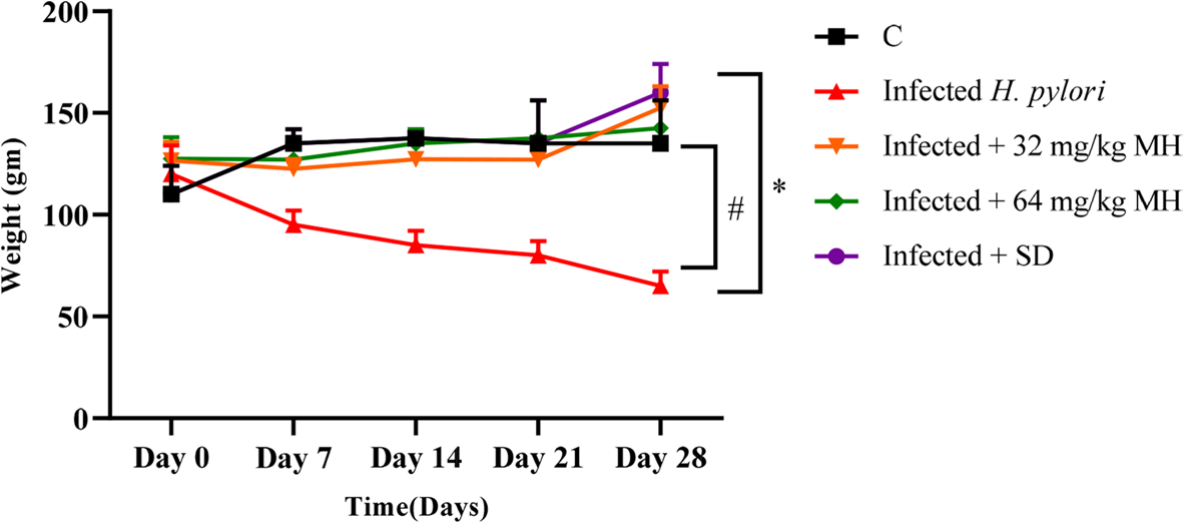
Determination of the variations in the weights of the animals in various examined groups (*n* = 10 rats per group; data are presented as means ± SEM). One-way ANOVA revealed a significant effect of treatment on final body weight (F(4,45) = 8.42, *p* = 0.0003). * *p* ≤ 0.05 compared to negative control; # *p* ≤ 0.05 compared to infected untreated group)

The change in weights of animals in the tested groups could be seen in (Fig. 3). There is a significant reduction (*P* ≤ 0.05) in the weights of animals infected with *H. pylori* relative to negative control group. While, a notable elevation (*P* ≤ 0.05) in the animals' weights in the treated groups with low, high dose of honey as well as the standard drug relative to infected group.

### Comparative testing for CFU count in stomach in examined groups

The bacterial count has been determined in the stomach of animals in the examined groups at various time points (Table 5). There is a significant rise (*P* ≤ 0.05) in the number of *H. pylori* in the infected group in proportional to rising of time points. Administration of various treatments resulted in significant reduction (*P* ≤ 0.05) by rising the time. It could be noticed that upon administration of 32 mg/kg of Manuka honey resulted a significant reduction (*P* ≤ 0.05) of CFU count by rising the time and reaching the maximal reduction at day 28. While, administration of either 64 mg/kg of Manuka honey or a standard drug resulted a significant reduction (*P* ≤ 0.01) of CFU count by rising the time and no detectable bacteria by culture at the end of the experiment.

**Table 5:** CFU count for *H. pylori* in different tested groups (*n* = 10 rats per group for in vivo data; Data are represented as means ± SEM; different letter above numbers in the same raw refers to significant difference where a: b *P* ≤ 0.05; a: c *P* ≤ 0.01; b:c *P* ≤ 0.05)

Treatments	Negative control	Infected *H. pylori*	Infected + 32 mg/kg MH	Infected + 64 mg/kg MH	Infected + SD
Time (days)					
0	0	1 × 10^6a^	1 × 10^6a^	1 × 10^6a^	1 × 10^6a^
7	0	1 × 10^7a^	1 × 10^4b^	0.5 × 10^4c^	0.4 × 10^4c^
14	0	2 × 10^8a^	1 × 10^2b^	0.3 × 10^2c^	0.2 × 10^2c^
21	0	3.1 × 10^9a^	35^b^	13^c^	9^c^
28	0	14 × 10^9a^	6^b^	3 ^c^	2^c^

### Histopathological examination of sections in various tested groups

**Fig. 4:**
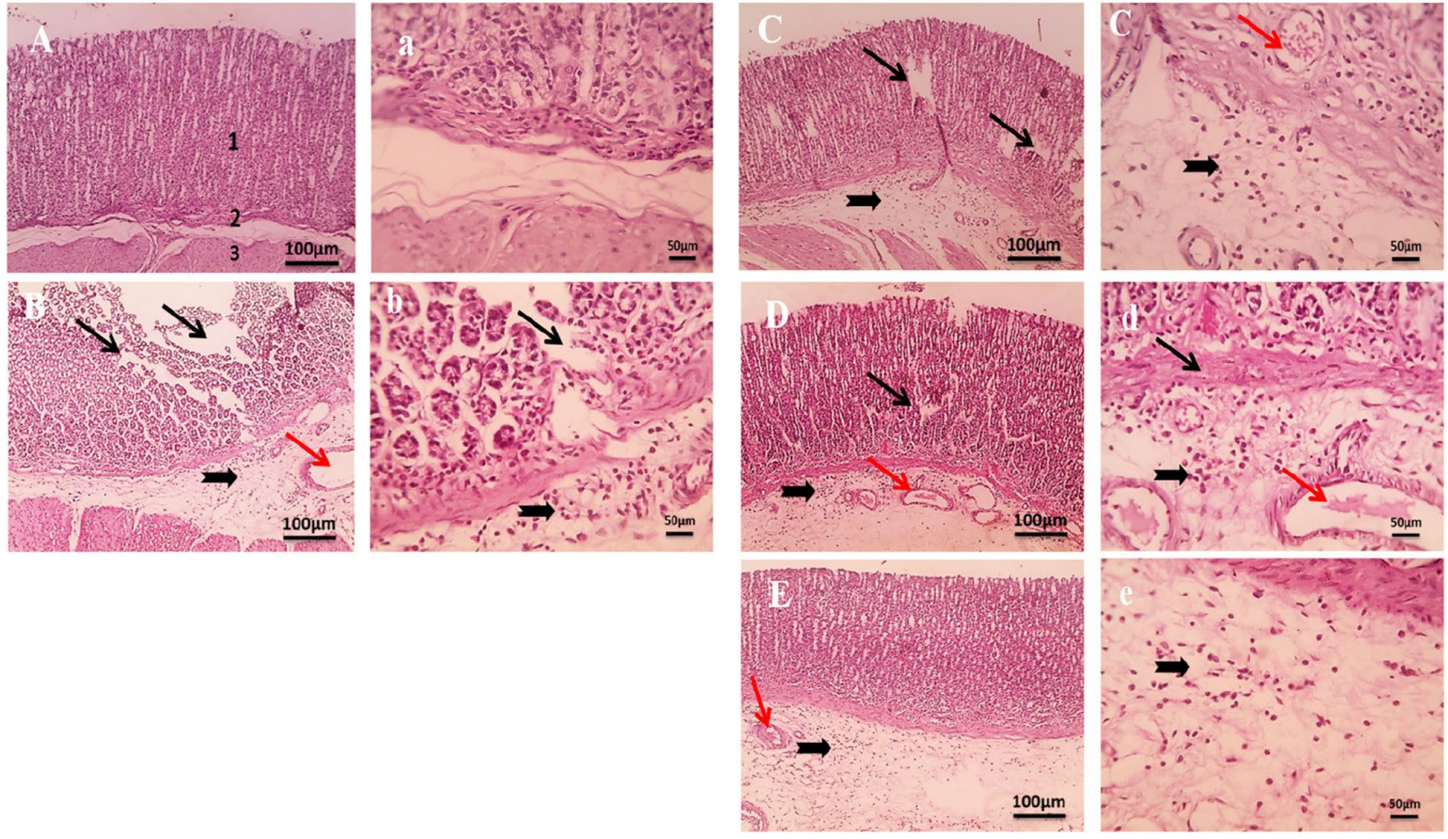
Representative photomicrographs of glandular gastric mucosa (H&E staining, × 100 bar 100 and × 400 bar 50) in control rats (**A**, a) showing normal intact layers mucosa 1, submucosa 2 and muscles 3 with no signs of inflammation. Glandular gastric mucosa in diseased rats infected with *H. pylori* (**B**, b) showing destructed mucosa, ulceration with marked loss in gastric glands (thin arrows). Glandular gastric mucosa in infected rats treated with honey at low dose (**C**, c) showing moderate damage and loss in gastric glands (thin black arrows), submucosal edema & inflammation (thick black arrows) with dilated blood vessels (red arrows). Glandular gastric mucosa in infected rats treated with high doses of honey (**D**, d) showing mild damage and loss in gastric glands (thin black arrows), submucosal edema & inflammation (thick black arrows) with dilated blood vessels (red arrows). Glandular gastric mucosa in infected rats treated with standard drug (**E**, e) showing normal mucosa and restored gastric glands, submucosal edema & inflammation (thick black arrows) with dilated blood vessels (red arrows). Abbreviations: VL, villous length; VW, villous width; H&E, hematoxylin and eosin

Hematoxylin and Eosin staining of the sections from various groups showed that the gastric wall in the negative control group has no signs of inflammation and a superbly preserved structure with intact muscular, submucosal, and mucosal layers (Fig. 4A, a). Additionally, the mucosa of rats infected with *H. pylori* shows severe pathological changes, such as ulceration, damaged epithelial lining, and a noticeable decrease in the number of stomach glands (Fig. 4B, b). However, the mucosa of sick rats given low-dose honey shows some recovery, but there is still considerable glandular atrophy, submucosal edema, inflammatory cell infiltration, and blood vessel dilatation (Fig. 4C, c). Additionally, the stomach tissue shows more obvious repair in diseased rats given high-dose honey, with only slight glandular loss, decreased submucosal edema and inflammation, and persistent but less noticeable vascular dilatation (Fig. 4D, d). Lastly, least, rats given standard drugs show little inflammatory alterations, reestablished stomach glands, and a largely recovered mucosal structure, albeit some submucosal blood vessel dilatation is still seen (Fig. 4E, e). Semi-quantitative scoring using the Updated Sydney System (Supplementary Table S2) confirmed the histopathological observations. The infected untreated group showed high scores for neutrophilic activity (2.9 ± 0.1), chronic inflammation (2.8 ± 0.2), glandular atrophy (2.8 ± 0.2), and surface epithelial damage (2.7 ± 0.2). Treatment with high-dose Manuka honey (64 mg/kg) significantly reduced these scores to 0.8 ± 0.2, 1.0 ± 0.2, 1.0 ± 0.2, and 0.9 ± 0.2, respectively (*p* < 0.001 for all comparisons), comparable to the standard drug group.

**Fig. 5:**
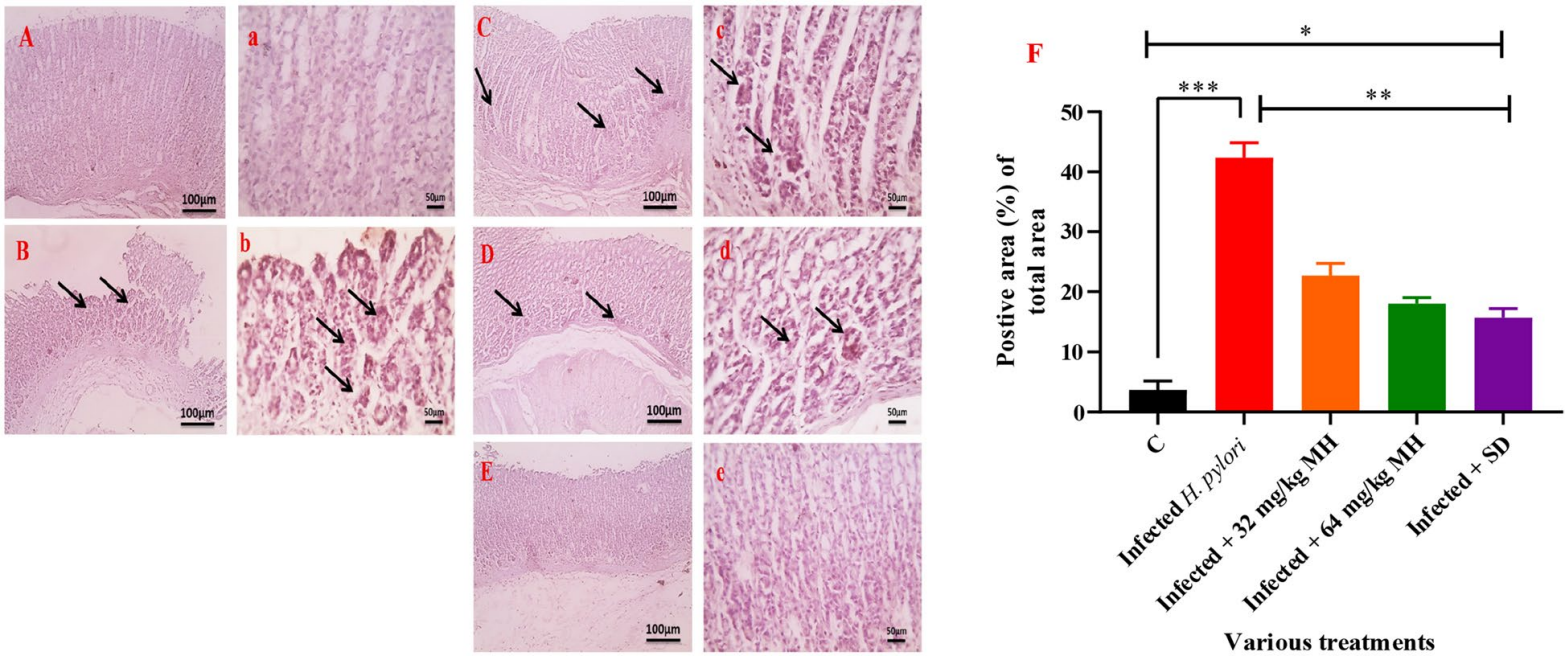
Representative photomicrographs of glandular gastric mucosa (IHC staining counterstained with Mayer's hematoxylin, × 100 bar 100 and × 400 bar 50) in control rats showing normal intact layers mucosa negatively stained for NF-kb (A, a). Glandular gastric mucosa in diseased rats infected with *H. pylori* showing marked positive brown staining in glandular cells for NF-kb in area of damage (thin arrows) (B, b). Glandular gastric mucosa in infected rats treated with honey low showing decreased positive brown staining in glandular cells for NF-kb in area of damage (thin arrows) (C, c). Glandular gastric mucosa in infected rats treated with honey high showing much less positive brown staining in glandular cells for NF-kb in area of damage (thin arrows) (D, d). Glandular gastric mucosa in infected rats treated with standard drugs showing negatively stained for NF-kb (E, e). **F** Statistical analysis to illustrated variations in the percentage of the positively stained area relative to area using ImageJ software (*n* = 10 rats per group for in vivo data; Data are presented as means ± SEM where * *P* ≤ 0.05; ** *P* ≤ 0.01; *** *P* ≤ 0.001 (**F**). Abbreviations: NF-κB, nuclear factor kappa B; IHC, immunohistochemistry

Immunohistochemical staining for NF-κB revealed clear differences among the experimental groups. Gastric mucosa from control rats showed intact mucosal architecture with negative NF-κB immunoreactivity (Fig. 5A, a). In contrast, *H. pylori*–infected rats displayed intense positive brown nuclear staining for NF-κB within the glandular epithelial cells, particularly concentrated in areas exhibiting structural damage. There is a dramatic rise in the intensity of the positively stained cells (*P* ≤ 0.001) relative to negative control (Fig. 5B, b). Rats infected with *H. pylori* and treated with low-dose honey demonstrated a reduction in NF-κB positivity, although staining was still detectable within the damaged regions. (Fig. 5C, c). Administration of high-dose honey produced a more pronounced effect, with markedly diminished NF-κB staining in the glandular cells of affected mucosal areas (Fig. 5D, d). Treatment with the standard drug regimen resulted in near-complete absence of NF-κB staining, comparable to the negative control group (Fig. 5E, e). There is a notable reduction in the intensity of the positively stained cells (*P* ≤ 0.01) relative to infected group.

### Evaluation for the variations for the inflammatory markers

**Fig. 6:**
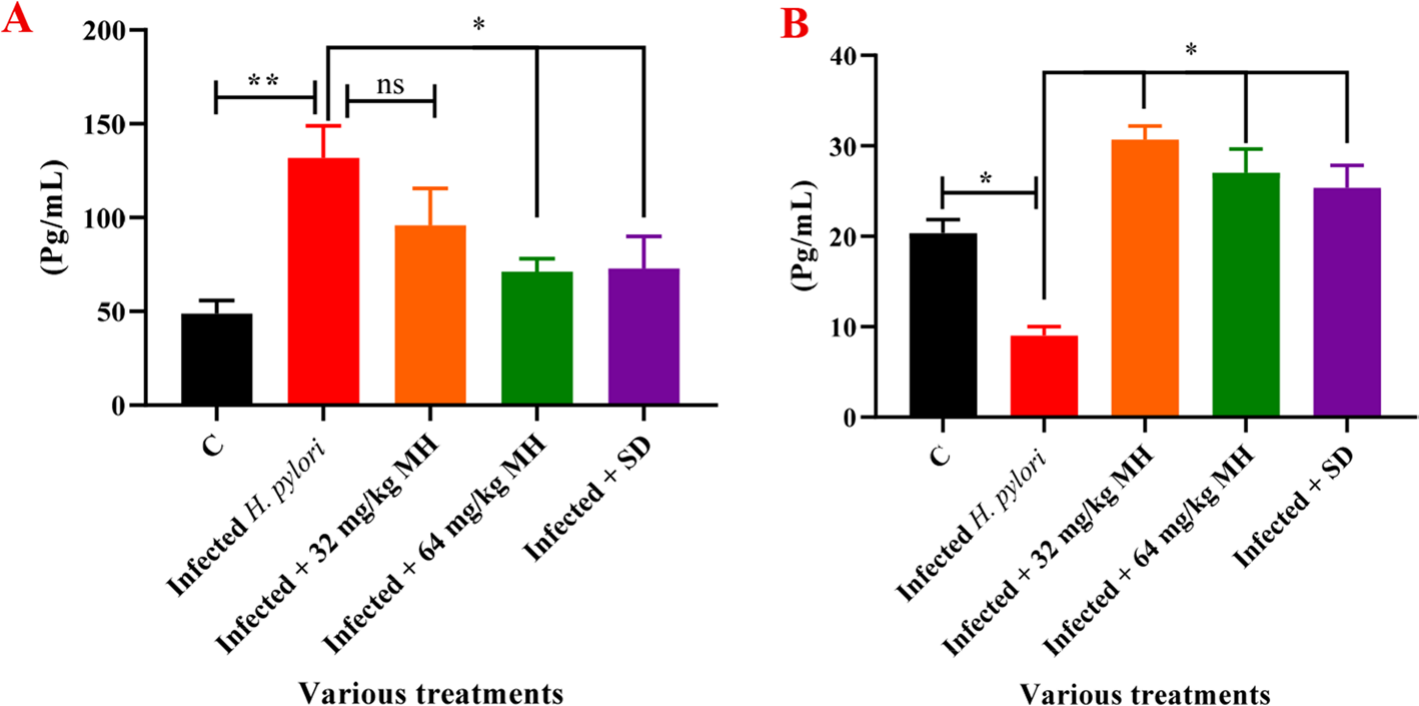
Statistical analysis for inflammatory mediators' levels in various examined groups (**A**) TNF-α (pg/mL), **B** IL-4. *n* = 10 rats per group; data are presented as means ± SEM. One-way ANOVA: For TNF-α, F(4,45) = 9.87,* p* = 0.0001; for IL-4, F(4,45) = 7.23, *p* = 0.0008. ** *p* ≤ 0.01, * *p* ≤ 0.05, n.s. = non-significant (*p* > 0.05)

Testing the change in the levels of proinflammatory marker (TNF-α) and anti-inflammatory mediator (IL-4) in various groups could be seen in (Fig. 6), There is a significant elevation (*P* ≤ 0.05) in TNF-α level in infected animals using *H. pylori* relative to control group. While, administration of 32 mg/kg of Manuka honey resulted in a notable reduction in TNF-α level in contrast to infected group. Furthermore, administration of 64 mg/kg of Manuka honey or standard drugs results in a significant reduction (*P* ≤ 0.05) in TNF-α level compared to infected group (Fig. 6A). On the other hand, IL-4 level had been significantly decreased (*P* ≤ 0.05) in infected group relative to negative control group. While, administration of 32 mg/kg or 64 mg/kg of Manuka honey or standard drugs resulted in a significant elevation (*P* ≤ 0.05) in IL-4 level in contrast to infected group (Fig. 6B).

### Elucidation for the changes for the oxidative markers

**Fig. 7:**
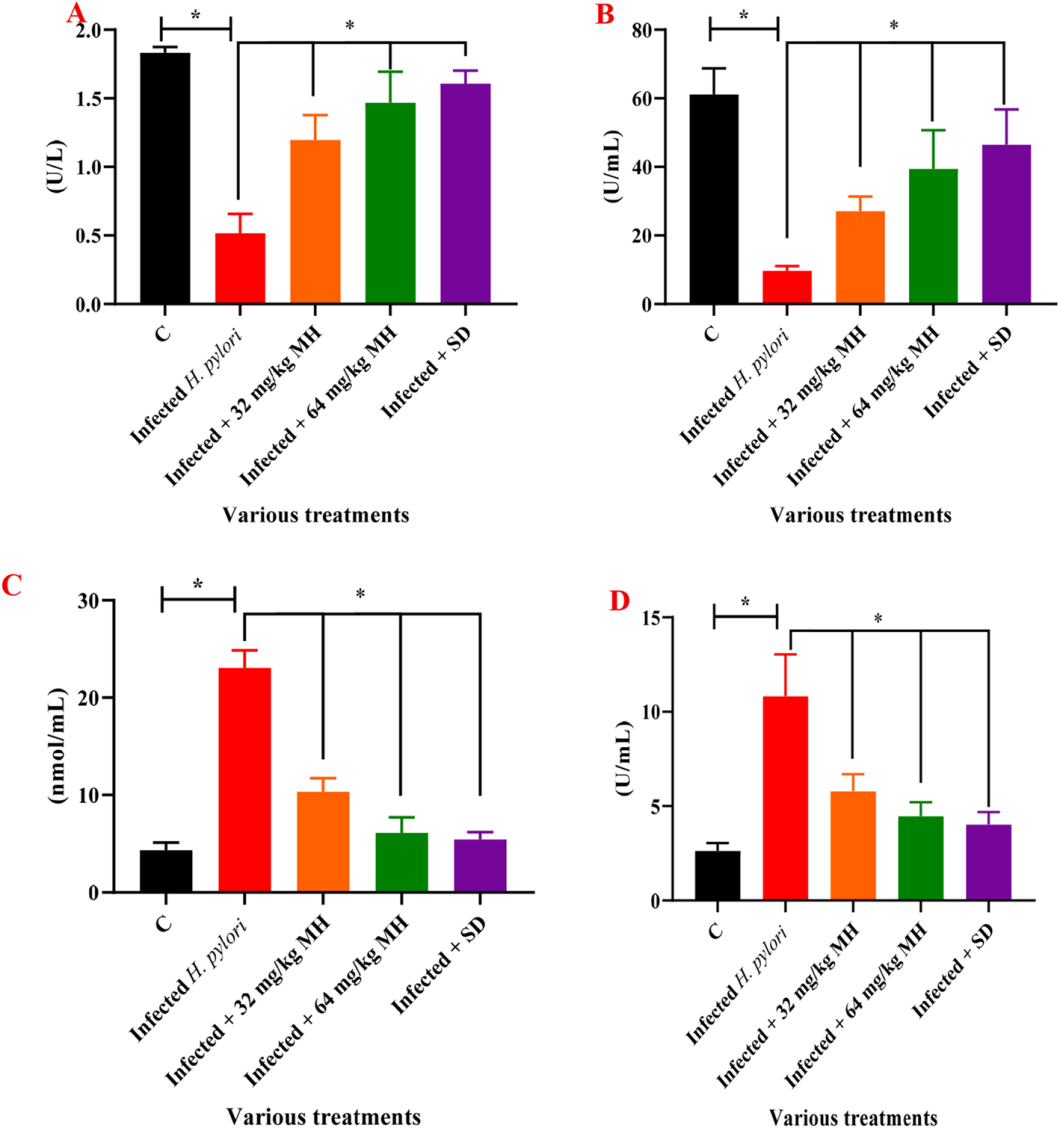
Statistical testing for oxidative markers levels in different tested groups (**A**) Catalase (U/L), **B** SOD (U/mL), **C** MDA (nmol/mL), **D** NO (U/mL) (*n* = 10 rats per group for in vivo data; outcomes are represented as means ± SEM; * *P* ≤ 0.05)

There is significant reduction (*P* ≤ 0.05) catalase (CAT) and superoxide dismutase (SOD) levels in the infected animals by *H. pylori* relative to negative control group**.** While, administration of 32 mg/kg or 64 mg/kg of Manuka honey or standard drugs resulted in a significant elevation (*P* ≤ 0.05) in oxidative markers levels in contrast to infected group (Fig. 7A, B). On the other hand, there is significant elevation (*P* ≤ 0.05) malondialdehyde (MDA) and nitric oxide (NO) levels in the infected animals by *H. pylori* relative to negative control group. While, administration of 32 mg/kg or 64 mg/kg of Manuka honey or standard drugs resulted in a significant reduction (*P* ≤ 0.05) in tested oxidative markers levels in contrat to infected group (Fig. 7C, D).

### Illustrations for the alterations for the biochemical markers

**Fig. 8:**
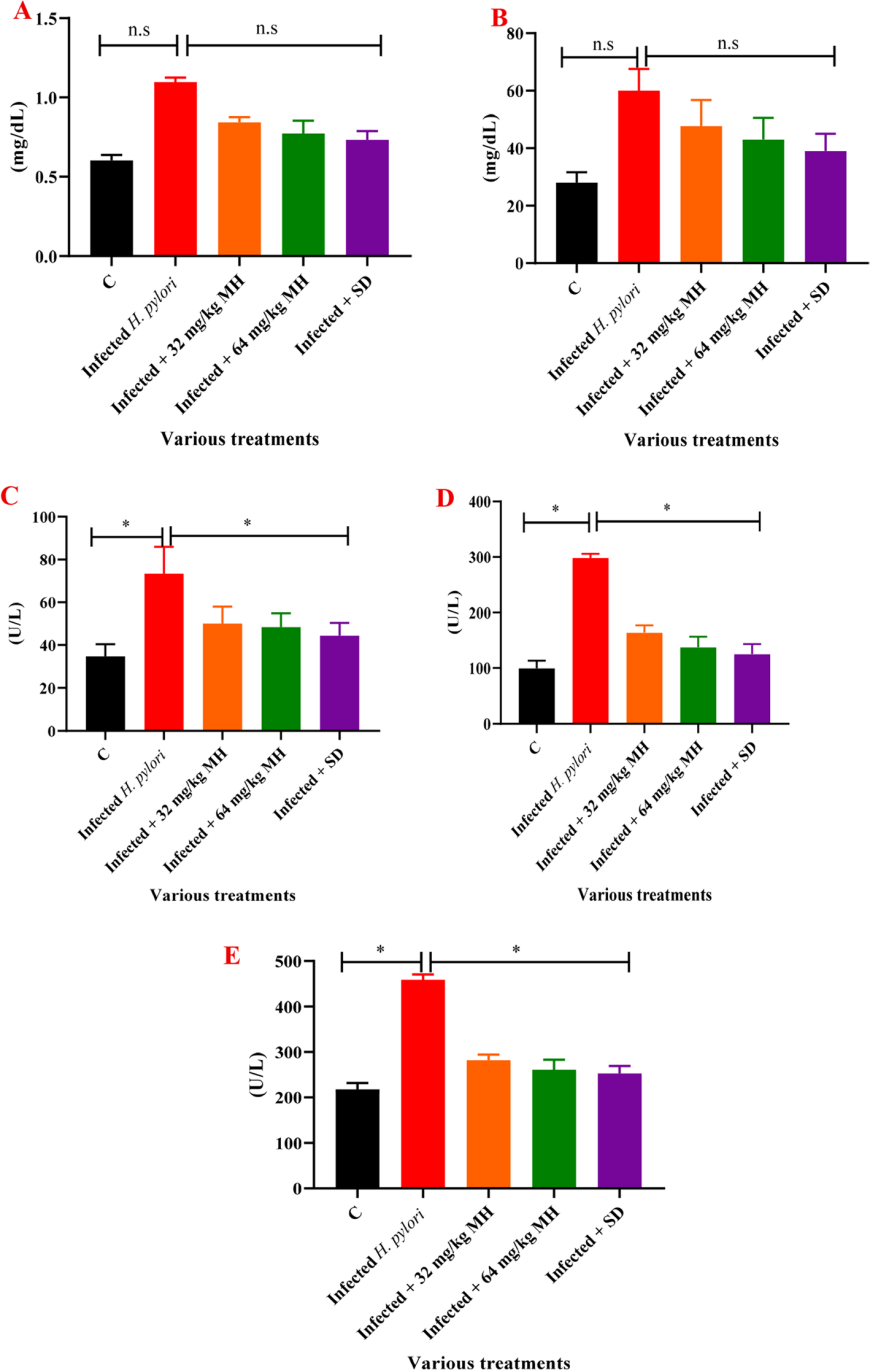
Statistical testing for renal and liver functions livers levels in different tested groups (**A**) Creatinine (mg/dL)**, B** BUN (mg/dL), **C** ALT (U/L), **D** AST (U/L), **E** ALP (U/L) (*n* = 10 rats per group for in vivo data; outcomes are represented as means ± SEM; * *P* ≤ 0.05)

There is a notable elevation in creatinine and blood urea nitrogen (BUN) levels in infected group relative to negative control group. However, administration of 32 mg/kg or 46 mg/kg of Manuka honey or standard drugs resulted in optimization of tested kidney functions levels in contrast to infected group (Figs. 8A, B). Furthermore, there is a significant elevation (*P* ≤ 0.05) in liver functions levels (AST, ALT, ALP) in infected group relative to negative control group. While, administration of 32 mg/kg or 46 mg/kg of Manuka honey or standard drugs resulted in a significant reduction (*P* ≤ 0.05) in liver functions levels (AST, ALT, ALP) in contrast to infected group (Figs. 8C, D).

## Discussion

The current investigation offers a thorough assessment of the phytochemical composition and in vitro anti-*H. pylori* function of Manuka honey extract, laying the groundwork for comprehending its therapeutic potential before the ensuing in vivo studies. This work demonstrates the kinetics of bacterial inhibition as well as the chemical components relevant for bioactivity by combining chromatographic profiling with many antibacterial assays. These results offer critical comparative standards for subsequent in vivo results and are vital for understanding how Manuka honey may has biological impacts within live systems.

Manuka honey is rich in a variety of phenolic and flavonoid components, according to HPLC studies, with the most prevalent phenolics being coumaric acid, cinnamic acid, and chlorogenic acid. These substances are well known for their potent antibacterial and antioxidant properties, which may help explain the biological activity seen in subsequent tests. The honey extract's bioactivity is further supported by the existence of seven flavonoids, especially chrysin, rutin, and kaempferol, which are known to damage bacterial metabolism and break microbial membranes [41, 42]. These polyphenols' coexistence raises the possibility of combined effects that could improve the effectiveness of antibacterial agents. Together, the chemical profile shows that Manuka honey has a large pool of bio-activities that are probably in charge of its later-observed anti-*H. pylori* qualities [43, 44].

The Manuka honey extract showed significant antibacterial properties versus *H. pylori*, with an inhibition zone a bit larger than the conventional medication, indicating its promise as a natural antibacterial agent. The MIC value (31.25 µg/mL) and MBC value (62.5 µg/mL) indicate bactericidal activity rather than bacteriostatic action. These findings are consistent with polyphenols' known ability to interact with microbial cell walls, disrupt enzymatic processes, and produce oxidative stress within microbes [45, 46]. The extract's equivalent activity to the normal medicine shows that it may have therapeutic use, especially in cases of resistance to antibiotics. Such in vitro efficacy presents a compelling case for investigating its efficacy in following in vivo studies [47].

Time-kill study demonstrated a time-dependent rise in decreased bacterial counts for both Manuka honey extract and the conventional medication, with no significant distinction across the two treatments throughout the 0–180-min period. This suggests that the extract has the potential of producing bactericidal effects at a rate in comparison to regular therapy. The gradual decrease in bacterial count shows that the honey's bioactive components exert ongoing antimicrobial impact, presumably through membrane rupture and suppression of bacterial multiplication [48]. The closeness in killing curves demonstrates Manuka honey's efficacy as a natural medicinal approach. These kinetics support the hypothesis that the extract's effect is not temporal but rather has a persistent bactericidal action, highlighting its potential importance for future in vivo studies [49].

The antibacterial mechanism of Manuka honey against *H. pylori* is primarily attributed to methylglyoxal (MGO), which exerts bactericidal effects through multiple pathways. MGO is known to form advanced glycation end-products (AGEs) on bacterial surface proteins, disrupting membrane integrity and interfering with flagellar motility, which is critical for *H. pylori* colonization of the gastric mucosa [41, 44]. Additionally, MGO induces oxidative stress within bacterial cells by depleting intracellular glutathione and inhibiting key metabolic enzymes such as glyceraldehyde-3-phosphate dehydrogenase [3, 50]. Importantly, the phenolic compounds identified in our HPLC analysis (coumaric acid, cinnamic acid, and chlorogenic acid) act synergistically with MGO. Phenolic acids disrupt the bacterial cell wall by chelating divalent cations (Mg^2^⁺, Ca^2^⁺) essential for lipopolysaccharide stability, thereby increasing membrane permeability and facilitating MGO entry into the bacterial cytoplasm [43–45]. This dual mechanism—membrane disruption by phenolics followed by intracellular damage by MGO—explains the rapid bactericidal effect observed in our time-kill assay (complete killing within 180 min) and the low MIC (31.25 µg/mL) comparable to conventional antibiotics. Furthermore, the concentration-dependent activity we observed (low-dose vs. high-dose honey) correlates with the MGO content (≥ 550 mg/kg), as higher MGO concentrations are associated with greater antibacterial potency [20, 44]. These mechanistic insights support the therapeutic potential of Manuka honey as an alternative or adjunctive treatment for *H. pylori* infection, particularly in the context of rising antibiotic resistance [51–54].

Beyond its direct antibacterial and anti-inflammatory effects, the physicochemical properties of Manuka honey may contribute to its gastroprotective efficacy against *H. pylori*. The high viscosity and osmotic activity of honey (due to its high sugar content, primarily fructose and glucose) create a viscous, adhesive barrier over the gastric mucosa, which prolongs contact time with the epithelial surface [12, 54]. This mucoadhesive property is particularly relevant for *H. pylori* therapy, as the bacterium resides deep within the gastric mucus layer and is partially shielded from water-soluble antimicrobial agents [16]. The hyperosmolar nature of honey may also draw fluid from the inflamed submucosa, reducing edema and improving tissue oxygenation [48]. Furthermore, the slow diffusion of honey through the mucus layer—compared to conventional aqueous antibiotic solutions—allows for sustained release of bioactive compounds (MGO and phenolic acids) into the gastric glands, potentially enhancing their accessibility to colonizing *H. pylori* [43, 44]. These physicochemical advantages, combined with the direct bactericidal and anti-inflammatory actions discussed above, position Manuka honey as a uniquely multifunctional therapeutic agent for *H. pylori* infection. Although not directly tested in this study, the complementary mechanisms of Manuka honey (membrane disruption by phenolics, intracellular damage by MGO, and anti-inflammatory/antioxidant effects) suggest potential synergistic interactions with conventional antibiotics. Future studies should investigate whether combining Manuka honey with clarithromycin or amoxicillin could lower the required antibiotic doses, reduce side effects, and overcome antibiotic resistance in *H. pylori* [51, 52].

The current findings on weight changes in *H. pylori*-infected rats are consistent with prior research showing that *H. pylori* infection causes metabolic stress, decreased appetite, and impaired nutritional consumption, all of which led to weight loss. Investigators found significant reductions in body weight after *H. pylori* infection [55, 56]. Supplementing with natural bioactive compounds, notably honey and polyphenol-rich extracts, has been proven to reverse infection-induced weight loss, which is consistent with our findings [57]. Manuka honey's capacity to restore body weight in our investigation is consistent with the findings of Almasaudi et al., [21] who illustrated that increased dietary intake and metabolic restoration in infected animals treated with honey, most likely due to its anti-inflammatory, antioxidant, and tissue-healing properties.

In terms of bacterial load, the gradual increase in CFU counts in untreated infected animals' correlates to *H. pylori* proliferation in the stomach mucosa, which has been extensively described. The significant reduction in bacterial burden observed after treatment with Manuka honey is consistent with previous research [19, 58] which demonstrated the powerful bactericidal properties of honey towards *H. pylori* in both in vitro and in vivo models. In our investigation, high-dose Manuka honey resulted in no detectable bacteria by culture by the conclusion of the trial, which is consistent with previous studies by Johnston et al., [3] who attributed honey's robust antibacterial effect to its high methylglyoxal and phenolic component content.

The histopathological changes seen in the *H. pylori*-infected group in this study are similar to those found in earlier experimental studies. Studies by Kao et al. [59] and Lanas and Chan [60] have shown that *H. pylori* colonization causes severe epithelial destruction, glandular loss, mucosal ulceration, and significant inflammatory infiltration. The profound restoration of gastric structure in honey-treated groups, especially when using high-dose Manuka honey, is consistent with the findings of Almasaudi et al., [21] who discovered that honey has strong mucosal healing capabilities due to its antioxidant, anti-inflammatory, and tissue-repairing effects. Our findings, which showed a reduction in edema, inflammatory cell infiltration, and vascular dilatation, add to the growing body of evidence that Manuka honey improves tissue regeneration and reduces infection-induced stomach damage. Furthermore, the near-complete mucosal regeneration seen with standard medications is consistent with the well-established efficacy of traditional triple therapy in treating *H. pylori*-induced gastritis as reported by Elbehiry et al., [6].

The immunohistochemistry findings for NF-κB support the observed histological improvements. *H. pylori* infection significantly boosted NF-κB activation, as seen in previous investigations by several research groups [22, 23] Damaged gastric epithelia showed substantial nuclear staining. This transcription factor is a key regulator of inflammation, and its strong activation is characteristic of *H. pylori*-induced gastric disease. Previous studies have shown that Manuka honey's phenolic contents and antioxidant effect can block NF-κB signaling, resulting in dose-dependent inhibition of NF-κB expression. Many investigators [21, 61] found that honey reduces NF-κB in gastric and inflammatory models, which supports our findings. The downregulation of NF-κB expression by Manuka honey may occur through both direct and indirect mechanisms. Directly, the phenolic compounds identified in our HPLC analysis—particularly chrysin, kaempferol, and coumaric acid—have been shown to inhibit the IKK complex (IκB kinase), thereby preventing phosphorylation and proteasomal degradation of IκBα, the inhibitory protein that retains NF-κB in the cytoplasm [41, 42, 62]. This allows NF-κB to remain sequestered in the cytoplasm, unable to translocate to the nucleus and activate pro-inflammatory gene transcription. Indirectly, the potent antioxidant activity of Manuka honey (evidenced by restored SOD and CAT levels and reduced MDA and NO) may suppress NF-κB activation by reducing intracellular reactive oxygen species (ROS), which are known upstream activators of the IKK pathway [23, 63]. Given that *H. pylori* infection induces significant oxidative stress in gastric epithelial cells, the reduction in ROS by honey's flavonoids and phenolic acids likely attenuates this pro-inflammatory signaling cascade. Thus, we hypothesize that both direct IKK inhibition by phenolics and indirect ROS scavenging contribute to the dose-dependent downregulation of NF-κB observed in our study. The conventional medication group experienced nearly complete normalization of NF-κB immunoreactivity, consistent with documented findings that eradication therapy effectively lowers inflammatory signaling pathways [64].

The changes in inflammatory markers found in this investigation are consistent with previous research on the immunological response to *H. pylori* infection. Yamaoka, [62] found that *H. pylori* significantly increase TNF-α levels via activating NF-κB and associated pathways. Manuka honey's significant reduction in TNF-α levels, especially at higher doses, aligns with the findings of Almasaudi et al., [21] who found that honey's antioxidant and immunomodulatory properties reduced inflammatory cytokines in gastric injury models. The observed rise in IL-4 in honey-treated animals also correlates with earlier studies showing that honey induces a shift onto an anti-inflammatory Th2-mediated response, as explained by previous reports [38, 65].

The findings on oxidative stress support Manuka honey's successful effect. The deficits in CAT and SOD in infected rats reflect the oxidative imbalance caused by *H. pylori*, as previously reported by Han et al., [23] who found that infection causes excessive ROS production and antioxidant depletion. The restoration of CAT and SOD levels after honey supplementation is consistent with the findings of El-Sawaf et al., [66] who attributed honey's antioxidative properties to its high flavonoid content. Similarly, the enhanced MDA and NO levels seen in infected rats are consistent with previous studies demonstrating increased lipid peroxidation and nitric oxide production during *H. pylori*-induced inflammation. Manuka honey's ability to drastically lower these indicators suggests strong radical-scavenging activity, similar with findings by Ahmed et al., [63].

The biochemical indicators also showed changes similar to those seen in previous studies [67]. Elevated creatinine, BUN, AST, ALT, and ALP levels in the infected group indicate systemic stress and secondary organ dysfunction caused by *H. pylori*, as supported by Abd El- Maksoud et al., [68] Manuka honey treatment resulted in considerable normalization of kidney and liver indicators, which is consistent with previously described hepatoprotective and nephroprotective capabilities. Meligi et al. [68] found that honey supplementation led to comparable improvements in biochemical indicators in oxidative stress models, which were attributable to decreased inflammation and increased antioxidant capacity. Collectively, these findings add to the expanding body of research indicating that Manuka honey has significant anti-inflammatory and mucosal-protective properties comparable to current pharmacotherapy.

Several limitations of this study should be acknowledged. First, only a single reference strain of *H. pylori* (ATCC 43504) was used; clinical isolates with varying antibiotic resistance profiles and virulence factors (e.g., *cagA* and *vacA* status) may respond differently to Manuka honey treatment. Second, the absence of multidrug-resistant clinical isolates limits the generalizability of our findings to real-world clinical settings, where resistance to clarithromycin and metronidazole is increasingly prevalent. Third, although the rat model is well-established for *H. pylori* infection, it does not fully replicate the chronic, relapsing nature of human infection or the complex gastric microbiome that influences treatment outcomes. Fourth, the 4-week treatment period, while standard for animal studies, is shorter than typical human eradication therapies (7–14 days of triple therapy followed by prolonged follow-up). Fifth, we did not assess the potential emergence of honey-resistant *H. pylori* strains, although resistance to honey's multiple bioactive components (MGO and phenolics acting via different mechanisms) is theoretically less likely than to single antibiotics. Sixth, the MGO content (≥ 550 mg/kg) and phenolic profile are specific to the commercial batch used (UMF 15 +); other Manuka honey products with different UMF grades (e.g., UMF 10 + vs. 20 +) may yield different results. Seventh, while our findings demonstrate clear therapeutic promise in an animal model, extrapolation to human clinical use requires further validation through controlled clinical trials with appropriate sample sizes and standardized honey preparations. Future studies should also investigate the effect of Manuka honey on *H. pylori* biofilm formation and urease activity, as these are key virulence factors (e.g.: *cagA*, *vacA*) contributing to treatment resistance.

## Conclusion and recommendation

This study demonstrates that Manuka honey exhibits strong antibacterial activity against *H. pylori*, comparable to conventional antibiotic therapy. Its high phenolic and flavonoid content contributes to both antibacterial and antioxidant properties. High-dose honey resulted in a substantial reduction (≥ 99.9%) of gastric *H. pylori* burden in vivo while also significantly improving histological characteristics. Manuka honey significantly reduced NF-κB activation, demonstrating its anti-inflammatory properties. It also improved oxidative equilibrium by raising CAT and SOD and decreasing MDA and NO concentrations. Enhancements in TNF-α and IL-4 levels demonstrate immunomodulatory capacity. Furthermore, Manuka honey reduced elevated liver and kidney biochemical markers associated with infection. These data indicate that Manuka honey provides multifaceted gastroprotection in an animal model, supporting its potential as a natural complementary therapy subject to further clinical validation. Further research, including controlled clinical trials in human subjects, is needed to confirm the clinical importance of these findings.

## Supplementary Information

Supplementary Material 1.

## Data Availability

The data supporting the findings of this study are available from the corresponding author upon reasonable request.
