# Supplementary material for: Manuka honey as a multifunctional therapeutic against Helicobacter pylori: from chemical profiling to in vitro potency and in vivo gastroprotective efficacy
Source: BMC Complement Med Ther. 2026 Aug 6;26:237. doi: 10.1186/s12906-026-05491-3 (PMC13445924; doi:10.1186/s12906-026-05491-3)
Supplement: Supplementary file 1 [file 12906_2026_5491_MOESM1_ESM.docx]

**Supplementary Table S1.** HPLC validation parameters for phenolic and flavonoid compounds.

| Compound | Retention time (min) | LOD (µg/mL) | LOQ (µg/mL) | Recovery (%) | R² |
| --- | --- | --- | --- | --- | --- |
| Chlorogenic acid | 5.0 | 0.08 | 0.24 | 96.2 | 0.999 |
| Cinnamic acid | 6.0 | 0.12 | 0.36 | 94.8 | 0.998 |
| Ellagic acid | 8.0 | 0.15 | 0.45 | 93.5 | 0.997 |
| Ferulic acid | 9.0 | 0.09 | 0.27 | 97.1 | 0.999 |
| Gallic acid | 11.0 | 0.05 | 0.15 | 98.3 | 0.999 |
| Coumaric acid | 13.0 | 0.10 | 0.30 | 95.6 | 0.998 |
| Syringic acid | 15.0 | 0.11 | 0.33 | 94.2 | 0.998 |
| Chrysin | 3.0 | 0.21 | 0.63 | 92.3 | 0.996 |
| Apigenin | 4.2 | 0.18 | 0.54 | 93.9 | 0.997 |
| Catechin | 6.3 | 0.07 | 0.21 | 97.8 | 0.999 |
| Kaempferol | 9.0 | 0.14 | 0.42 | 95.1 | 0.998 |
| Galangin | 10.3 | 0.19 | 0.57 | 93.2 | 0.997 |
| Luteolin | 12.8 | 0.13 | 0.39 | 96.5 | 0.998 |
| Rutin | 14.8 | 0.16 | 0.48 | 104.7 | 0.997 |

**Supplementary Table S2.** Semi-quantitative histological scoring of gastric mucosa according to the Updated Sydney System. Data are presented as mean ± SEM (*n* = 10 rats per group). Scores: 0 = normal, 1 = mild, 2 = moderate, 3 = marked. Statistical analysis: One-way ANOVA with Tukey's post-hoc test. Different superscript letters (a, b, c, d) within the same row indicate significant differences at *p* < 0.05.

| Parameter | Negative control | Infected untreated | Infected + 32 mg/kg MH | Infected + 64 mg/kg MH | Infected + SD | F-value | *p*-value |
| --- | --- | --- | --- | --- | --- | --- | --- |
| Neutrophilic activity | 0.0 ± 0.0^a^ | 2.9 ± 0.1^b^ | 1.8 ± 0.2^c^ | 0.8 ± 0.2^d^ | 0.6 ± 0.1 ^d^ | 98.45 | < 0.0001 |
| Chronic inflammation | 0.0 ± 0.0 ^a^ | 2.8 ± 0.2 ^b^ | 1.9 ± 0.1 ^c^ | 1.0 ± 0.2^d^ | 0.8 ± 0.1 ^d^ | 87.32 | < 0.0001 |
| Glandular atrophy | 0.0 ± 0.0 ^a^ | 2.8 ± 0.2 ^b^ | 1.7 ± 0.2 ^c^ | 1.0 ± 0.2^d^ | 0.7 ± 0.2 ^d^ | 79.56 | < 0.0001 |
| Surface epithelial damage | 0.0 ± 0.0 ^a^ | 2.7 ± 0.2 ^b^ | 1.6 ± 0.1 ^c^ | 0.9 ± 0.2^d^ | 0.7 ± 0.1 ^d^ | 82.14 | < 0.0001 |

Statistical summary: One-way ANOVA revealed highly significant differences among groups for all four parameters (*p* < 0.0001). Tukey's post-hoc test showed that high-dose Manuka honey (64 mg/kg) and standard drug (SD) groups were not significantly different from each other (*p* > 0.05) for all parameters, but both were significantly different from the infected untreated group (*p* < 0.001) and low-dose honey group (*p* < 0.01).
